# The European Cystic Fibrosis Society Patient Registry: valuable lessons learned on how to sustain a disease registry

**DOI:** 10.1186/1750-1172-9-81

**Published:** 2014-06-07

**Authors:** Laura Viviani, Anna Zolin, Anil Mehta, Hanne Vebert Olesen

**Affiliations:** 1 Department of Clinical Sciences and Community Health, University of Milan, Milan, Italy; 2 Division of CVS and Diabetes, Ninewells Hospital and Medical School University of Dundee, Dundee, UK; 3 Pediatric Department, Aarhus University Hospital, Aarhus, Denmark

**Keywords:** Cystic fibrosis, Disease registry, Patient registry, Database

## Abstract

**Background:**

Disease registries have the invaluable potential to provide an insight into the natural history of the disease under investigation, to provide useful information (e.g. through health indicators) for planning health care services and to identify suitable groups of patients for clinical trials enrolment. However, the establishment and maintenance of disease registries is a burdensome initiative from economical and organisational points of view and experience sharing on registries management is important to avoid waste of resources. The aim of this paper is to discuss the problems embedded in the institution and management of an international disease registry to warn against common mistakes that can derail the best of intentions: we share the experience of the European Cystic Fibrosis Society Patient Registry, which collects data on almost 30,000 patients from 23 countries.

**Methods:**

We discuss the major problems that researchers often encounter in the creation and management of disease registries: definition of the aims the registry has to reach, definition of the criteria for patients referral to the registry, definition of the information to record, set up of a data quality process, handling of missing data, maintenance of data confidentiality, regulation of data use and dissemination of research results.

**Results:**

We give examples on how many crucial aspects were solved by the European Cystic Fibrosis Society Patient Registry regarding objectives, inclusion criteria and variables definition, data management, data quality controls, missing data handling, confidentiality maintenance, data use and results dissemination.

**Conclusions:**

We suggest an extensive literature research and discussions in working groups with different stake holders, including patient representatives, on the objectives, inclusion criteria and the information to record. We propose to pilot the recording of few variables and test the applicability of their definition first. The use of a shared electronic platform for data collection that automatically computes derived variables, and automatically performs basic data quality controls is a good data management practice, that also helps in reducing missing data. We found crucial for success the collaboration with existing national and international registries, cystic fibrosis organisations and patients’ associations.

## Background

A disease registry is a paper list or an electronic database containing information on the characteristics of a population affected by a given disease [1,2]. Disease registries have become essential for the investigation of chronic diseases thanks to their potential to epidemiologically describe the natural history of the disease. They are particularly useful in rare diseases, such as cystic fibrosis (CF), where important research questions cannot be answered without large multicentre studies because of the limited number of patients followed by individual CF centres.

The importance of disease registries has been acknowledged also by EUCERD [3], an EU Committee of experts in rare diseases that discusses policies and recommends activities in collaboration with the EU Commission and Parliament and the Council of Ministers. Many international projects, such as EPIRARE [4] and RD-CONNECT [5], promoting international registries have also been funded by the EU.

The institution and long term management of disease registries are not trivial challenges, since many key hurdles need to be overcome. To avoid the garbage in, garbage out phenomenon, the first step requires the definition of patients’ inclusion/exclusion criteria: it is vital to determine whether the registry records only confirmed cases, based on a set of pre-defined criteria, or whether it is open to all subjects with a suggestive set of symptoms or signs. The clinical and epidemiological questions the registry has to address and the set up of the information system depend on these criteria. The difficulty in addressing most of the hurdles is magnified in an international setting, where agreements on data definition are crucial to ensure uniformity of data collection across the participating countries.

Many registries begin on a voluntary and unfunded basis, being championed by single enthusiasts, but an effective disease registry requires sustained funding, adequate manpower and an efficient organisational structure to achieve its main purpose: to describe the clinical status of patients to foster care improvement [6,7].

The aim of this paper is to discuss the problems embedded in the institution and management of an international disease registry, to warn against common mistakes that can derail the best of intentions. The experience gained in the establishment and maintenance of the European Cystic Fibrosis Society Patient Registry (ECFSPR), that collects data from individual CF centres and national CF registries from Europe and the neighbouring countries, can be used to show the hidden dangers of disease registries. In this paper we provide examples on how several crucial aspects were solved by the ECFSPR in the areas of objectives, inclusion criteria and variables definition, data management, data quality controls, missing data handling, confidentiality maintenance, data use and results dissemination.

Prior to the establishment of the ECFSPR, a pan-European registry originating from a database set up to monitor a clinical trial on a respiratory medicine was used to collect data from approximately 10,000 patients in 9 countries. This registry was funded by F. Hoffmann-La Roche Ltd, and was named Epidemiologic Registry of Cystic Fibrosis [8]. With the termination of funding in 2002, it was decided by CF specialists across Europe, members of the European CF Society (ECFS), to set up an independent registry with clear objectives. At that time several national registries already existed, each built on separate proprietary platforms, whereas the majority of European countries did not have a CF registry in place, or had registries based on one-centre only. In 2003, under the auspices of ECFS, a working group of representatives of existing national registries was appointed to set up this new registry. A pilot study collected data from seven national registries (Belgium, Denmark, France, Ireland, Italy, Russia and Sweden), using a simple electronic spreadsheet. This provided a precious starting point for data collection, but also showed that different definitions were separately developed by each national registry, and even if specifications on data formats and coding were given, these were not always followed by data contributors. This aspect, along with the need for a tool for data entry for countries without a CF registry, led to the re-evaluation of the data collection system. A registry steering group was created, in charge of setting up the structure of the ECFSPR, defining its milestones and its roadmap.

The first turning point came with the EU funded project EuroCareCF, that in cooperation with the ECFSPR steering group set up patient consent forms and collected demographic data from 35 countries, thereby laying the foundation for expansion of the ECFSPR outside the existing national registries [9]. The second and probably most important turning point came with the support from the ECFS Board who decided to expand the financial support allowing appointment of professional staff (coordinator, helpdesk, statisticians) and lately the development of a bespoke software building on the experience of previous data collection and software.

## Methods

In this section we introduce the major problems that researchers often encounter in the creation and management of an international disease registry. Many critical aspects that we had to face in the activation of this international registry can be found also in the implementation of national/local disease registries and they can be faced in a similar way.

### Definition of the objectives and of the population under study

A registry is not an end in itself; it is rather a tool to reach predetermined objectives, such as enhancement of knowledge of the epidemiology of the disease under study, evaluation of different diagnostic and therapeutic protocols, evaluation and planning of public health resources, creation of health indicators to evaluate the disease burden and the efficacy of the care [10-12].

The use of registry data is effective and efficient only if there is a clear definition of the objectives the registry has to reach and a tracking of the outcomes is carefully planned. Stepping beyond these agreed objectives could be problematic especially during the set up phase, because the community’s expectations might become unachievable in the time frame of the available funding. In this phase, estimation of the observation time needed to obtain the results is a key factor, especially for rare diseases, due to the risk of having to wait for years before an adequate number of patients or outcomes are recorded. In a later phase, when core functions are running smoothly, objectives can be re-evaluated. Objectives should also be shareable by the patients whose data are collected in order to obtain their consent to process their data.

Ideally, a disease registry should only contain data on patients diagnosed with the disease of interest. This apparently trivial aspect is the core of data collection, because it determines the uniformity of population under study and it explicitly defines the data to be recorded. For this reason, it is crucial to set up an unequivocal definition of case, which rests upon a general agreement on the diagnostic criteria [13]. These criteria may be subject to change over time: often, the change of diagnostic practices due to advances in knowledge of the disease and advances in scientific technology change the inclusion criteria to registry referral. The key aspect is to keep an audit trail of these changes.

### Definition of what to measure and how to do it

In the planning of a disease registry, it is necessary to decide which aspects of the disease are to be recorded and identify their appropriate indicators. Enthusiasm often leads to substantial overestimation of the amount of information necessary to record: it is easy to fall into the mistake of recording far too much information than what is actually needed to answer the questions the registry has to address. This is a highly inefficient approach in terms of time needed to retrieve the data and to register them into the database, because ultimately the information will not be used. The amount of information recorded is a trade-off between the researchers’ needs and the requirement to keep the registry easy to handle [10]. It is therefore advisable in the planning phase to clearly define objectives and to agree on which information has to be recorded. These tasks, if well conducted, make data collection more efficient and avoid frustration in people who enter the data if the information is not used and frustration in statisticians who analyse empty databases. For some rare diseases, the information to be recorded may be difficult to target because the disease is not yet fully understood. It is therefore advisable in the planning phase to select information to be recorded that is agreed in literature, being aware that new information could be added in the future.

Definitions for registries have to reflect what is obtainable during daily clinical work at various centres, and in case of international registries, across various countries. Strict definitions based on well-defined clinical and para-clinical aspects, as are often implemented in clinical trials, are often not applicable in the daily clinic, making compromises necessary. Unless the data collected are useful to the clinician or other ways of data collector reward (including economic repayment) many registries can fail at this step.

### Data management and data quality controls

To reach its aims, an international patient registry has to set up an efficient data management system, preferably automated, that accommodates both national registries’ and individual centres’ needs, coordinating the work of database managers, statisticians, epidemiologists, and covering many aspects, such as data collection, data quality controls, error correction, data analyses and reporting. A limitation to smooth data streaming for an international patient registry is that national data registries (or individual centres) often change their data acquisition systems and any subsequent data sending to the international registry requires an agreement between all parties involved to prevent rupture of the data stream or unwelcome changes to it.

Data quality control is one of the core data management activities of a disease registry and it is probably the most important aspect because the quality of the data, together with efficiency of data management, inevitably affects the quality of research [13]. For this reason, it is vital for a registry to have accurate data quality controls and efficient data processing systems in place.

### Handling of missing data

Missing data are the bane of researcher’s lives because they can reduce the precision of the estimates and may lead to biased results. Missing data are a widespread problem: in many registries, although there is an adequate completeness level in the demographic data, there is questionable completeness for clinical follow-up data [14]. Although there are statistical techniques to address missing data, it is always preferable to prevent information loss, therefore it is essential to understand the mechanisms that cause it and avoid missing data as much as possible [10,13].

### Maintaining confidentiality of registry data

The binding element to legally collect data is to obtain the patient consent to process their data. In order to reassure the patients to increase their willingness to give their consent, it is crucial to ensure that patient’s identification and data security measures are state-of-the-art, particularly when dealing with electronic data, that such measures meet the data protection legislation, and that they are described and available to the patients. The collection of data into national registries and their potential export into an international registry must be approved by the data protection authority in the country of residence of the patient. An international registry data collection must itself be approved by the data protection authority of the country of the data controller. The key aspect is that the data sender must be legally allowed to send the data and the central registry must be legally allowed to process the data. The use of data must be described in data protection applications and in the patient consent information sheets; and it is important that the information given is detailed, but it also has to accommodate practical changes during the course of the registry life: revised applications for the data protection authorities in case of e.g. a change of data processor can be easily made, but obtaining the patient consents from all patients within a short time interval might be impossible. The registry needs to keep up to date with changing data legislation, nationally and internationally; this is an important challenge across Europe as new regulations are about to be enacted [15].

### Dissemination of the results and use of data

Timely dissemination of results and appropriate use of data collected are the key elements for achieving the registry primary objectives: enhance knowledge of the disease under study and promote research. The objectives of a disease registry also allow use of data for identifying patients eligible for clinical trials, e.g. patients with a rare genotype. These patients are of course not to be contacted directly by the company conducting the trial, but via their care giver, so anonymity is withheld until the patient consents to participate in the trial.

## Results

The results section presents the solutions to the hurdles described in the methods section, as implemented by the ECFSPR. The critical aspects and the solutions are summarised in Table 1.

**Table 1:** Overview of critical aspects when setting up a registry and the solutions implemented by European Cystic Fibrosis Society Patient Registry

**Critical aspects**	**Solution**
**Definition of the objectives of the registry**	Discussion on the objectives in a working group involving different stakeholders, including patient representatives
**Definition of the population under study**	
Definition of inclusion criteria	Extensive literature research, retrieval of necessary information from existing registries, harmonisation of criteria made by a working group, adoption of an operational definition that could be used as inclusion criteria for the registry purposes
Assessment of whether patients registered meet the inclusion criteria	Ideally, recording of all the information necessary to check diagnosis, but, operatively, assessment delegated to the data contributors who have to confirm that the inclusion criteria are met
**Definition of what to measure and how to do it**	
What to measure	Review of literature and discussion on variables definitions in a small working group of experts
How to measure	Start data collection of few variables and test with a pilot study the applicability of their definition
	If the definition used is not the same across countries:
	• try harmonisation by making the definition more generic
	• involve stakeholders to discuss change of definitions and agree on a shared definition
	• if definitions can be assimilated, report differences of definitions in the publications as caveats
**Data management and data quality controls**	
Data management	Shared electronic platform for data collection with automatic computation of derived variables, allowing both direct data entry and remote data upload.
	Use of technology (such as XML) that ensures that required data format and coding is used.
Data quality controls	Automatic and immediate data quality controls on entering (plausible ranges, intra-record data coherence, and consistent information across years.)
	Use of drop-down menus with fixed input possibilities (e.g. yes/no/unknown)
	Agreed controls with national registries in order to avoid duplication of identical data quality control processes.
	Use of refined data controls based on age-and-sex-specific reference values
	Set up of a data error procedure that uses a software that automatically warns and points the user to the data to correct
**Handling of missing data**	User-friendly software and useful feedback to contributors to encourage data entry
	Clear definitions, but attainable in daily clinical practice
	Unequivocal exhaustive variable coding with no pre-set values
	Avoid the use of tick boxes that code missing answers and negative answers the same way
	Working with existing registries to accommodate definitions
**Maintaining patient confidentiality**	Separate storage of encrypted personal data and anonymous centre numbers
	Pseudo-anonymisation to allow contact with centre for error correction
**Dissemination of data**	Code of conduct document concerning publication rights, authorship and data access – preferably set up very early in the process

### Definition of the objectives and of the population under study

#### Definition of the objectives

During the set up meetings of the first working group of the ECFSPR, the objectives of the registry were thoroughly discussed and agreed, as stated in the ECFSPR website and in patient consent [16]: “The purpose of the ECFS Patient Registry is to measure, survey and compare aspects of cystic fibrosis and its treatment in the participating countries, thereby encouraging new standards of dealing with the disease, to provide data for epidemiological research and to identify special patient groups suitable for multi-centre trials. The information will facilitate long-term planning of health expenditure allocations and developing pan European support systems”. When defining the objectives it is important to include all stake-holders in the process. For a patient registry relying on the patients acceptance of their data being collected, we found it crucial to involve patient representatives very early in the project; for this reason we cooperated closely with CF Europe, the European CF patients’ organisation, who appointed two of their associates as members of the steering group, one of whom is also member of the executive committee.

#### Definition of the population under study

Due to the international nature of the ECFSPR, the first concern was to ensure that the registry collected data from patients meeting uniform inclusion criteria across countries. A working group was set up with the aim of defining the inclusion and exclusion criteria for CF patient referral to the ECFSPR. This was a small group, whose tasks included extensive literature research, retrieval of necessary information from CF registries representatives and database managers, and harmonisation of criteria.

The diagnostic criteria for CF are not internationally agreed and, often, their verification is not strictly performed in clinical practice, mainly due to costs. For example, sodium and chloride concentrations in an agreed sweat test protocol are considered gold standards for the diagnosis of CF [17], but a quicker method of estimating sweat chloride (conductance) was introduced, even if it has never been recognized as equivalent to the concentration measurements [18]. However, this method is widely used as a screening tool and may be the only sweat test performed on a patient. Moreover, improvements in DNA analysis resulted in the diagnosis of both patients on whom sweat test was never performed (e.g. because two known disease causing mutations were found by DNA analysis) and patients with clinically milder forms of CF or CF-like syndromes, opening the debate in the international scientific community about the definition of CF [19].

The ECFSPR therefore adopted an operational definition that could be used as inclusion criteria for the registry purposes. The fundamental aspect in fact is that the population under study is uniform in terms of inclusion criteria, even if the official clinical definition might not be met, because this does not have an impact on research and appropriate subgroups can be selected for further study.

Ideally, the ECFSPR should record all the information necessary to assess whether the patients registered meet the inclusion criteria or not. However, the level of detail of information needed resulted in a lot of missing data from the pilot data collection, especially from the national registries, that often did not record such detailed information. The verification of the inclusion criteria was therefore delegated to the data contributors, who had the opportunity to check the clinical notes in a more efficient and timely way, and the ECFSPR data collection requires each participating country/centre to confirm, for each patient referred to the ECFSPR, that the inclusion criteria are met. The data senders therefore take responsibility on the appropriateness of referral, and no verification of the criteria is performed by the ECFSPR.

### Definition of what to measure and how to do it

#### Definition of what to measure

In the ECFSPR experience, the task of defining variables was particularly complex and time-consuming, due to the international nature of the registry and the fact that many countries had already established national registries using internally-chosen definitions. A small group of people was put in charge of revising the literature about the definition of critical variables and discussing the implementation and the adaptation of clinical definitions. The intention of this working group was to collect a limited set of key variables that would make it possible to achieve the ECFSPR objectives.

We first undertook the pilot data collection from the existing national registries to estimate the feasibility of collection of such variables. The participants were asked to send information for 49 variables. The proportion of missing data varied according to the type of information requested: basic demographic data such as gender, year and month of birth were reported on all patients, whereas more detailed social and clinical information such as marital status or use of continuous inhaled antibiotics was missing for almost all patients. This pattern was mostly due to some national registries not recording information on the variables required by the ECFSPR. Based on this collection, the definitions group put forward a revised set of variables for the collection of data for years 2004/2005.

#### Definition on how to measure

The ECFSPR could not always follow the golden rule according to which the choice of indicators should fall on those easy to observe and with unanimous definition. Often the best indicator was not the easiest one to observe: in this case, it was not included in the registry, due to the high risk of having a lot of missing data, obtunding the efforts of data collection. Particularly useful was the approach to have in the database few variables and test with a pilot data collection the applicability of their definition. Also, if the best indicator has different interpretations in the scientific community, there is the risk of putting under the same roof different quantities, making interpretation of results impossible. Some case examples are:

•Medication: asking quantitative information on the dose of pancreatic enzymes taken by CF patients is clinically useful and it would be highly informative, but the intake of this medication is often variable on a meal to meal basis and estimating a “daily intake value” (or a “yearly intake value”) would be extremely difficult. The definitions working group felt it was appropriate to replace this information with a more generic “use of pancreatic enzyme ever in the follow-up year”;

•Complications: an important prognostic factor in CF is chronic infection with *Pseudomonas aeruginosa*. Several definitions of chronic infection have been used in daily clinical care as well as in publications. The most commonly used is the Leeds definition [20] or a modification of this. However, this definition requires several sputum cultures per year, and patients who were diagnosed with chronic infections for a long time and on continuous inhalation therapy may not fulfil the strict criteria, although they would still be classified as chronically infected by their care givers. Therefore, the definition on diagnosis of chronic infections for the ECFSPR purposes had to be an operational definition [21] that allowed discrimination only between patients with chronic infection from those patients without infection and/or with intermittent infection.

Collecting about 85% of its data through national registries, which have already collected data from the CF centres following the national registry definitions, not necessarily the same as those of the ECFSPR, is another challenge. Whenever possible, we used a definition that would comply with most national registries. However, some of them could not be harmonised and for those the national registries have chosen either to report this variable as missing (e.g. one registry records only “*Pseudomonas aeruginosa* cultured this year” with no chronicity defined), or to change their definition to comply with the ECFSPR one. For example, several countries are now collecting the best value of FEV1 of the year, as required by the ECFSPR, instead of last of the year or the value registered at the annual assessment. If definitions are not the same across countries, but can be assimilated, this is reported in methods sections of manuscripts and written in notes appended to tables and graphs.

### Data management and data quality controls

#### Data management

**Figure 1:**
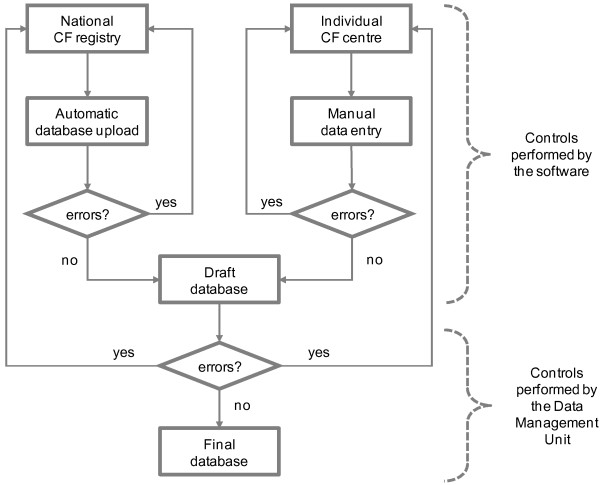
Flow-chart of data collection and data quality controls of European Cystic Fibrosis Society Patient Registry.

Figure 1 schematically shows how the ECFSPR data collection is organised. In order to provide a common platform for data collection, bespoke software was developed. It is composed of two tools: one for data upload from national registries and one for manual data-entry for countries that do not have a national database. In both cases, the data are stored on a central server, located in secure premises. The current data-entry software, which has been used for 3 years of data collection, has some limitations and new software is currently being rolled out across Europe, although the data flow structure at the core of its functioning has not changed.

##### Acquisition of data

The data upload from national registries is performed through the Extensible Mark-up Language (XML), a strategy adopted by the ECFSPR in 2010 to overcome the inefficiency in the coding of data from national registries. In fact, this choice of language forces the variables formats and specifications to be met, saving a lot of data management time to the ECFSPR Data Management Unit (DMU). Once the national registries have prepared the data extract from their database according to the agreed definitions and coding, they upload the XML file they created, and a two-step system validates the file according to a procedure described in the next section. The use of the XML procedure has as big limitation the need of an IT-specialist to create the XML file from the data extract made by the national registry data manager. In the ECFSPR experience, this aspect was a bigger obstacle than anticipated, both practically and psychologically. This made us rethink the upload process in the development of the new data-entry software, focusing on user-friendliness and ease of use without compromising on coding requirements. The new data-entry software allows uploading data files in different formats in addition to XML (such as comma-separated values, and common formats that are easily originated from the national databases) and controls on data coding and value ranges are performed on these files.

The manual data-entry is performed as follows: the software sends the patients’ data, except the identifiers, to a web server. The data are anonymised through the creation of a randomly-generated code. The identifiers, like name and full date of birth, are stored in an encrypted form on a server, but only the centre holds the key to de-crypt the data. The rest of the database is also stored in an encrypted format and it is password-protected. Some data-quality controls are automatically performed by the software, which warns the user by means of flagged fields whenever discrepancies are found and when items are left blank. Further details on automatic data quality controls are described in the next section. One limitation of the current software is that the non-anonymous data are stored at the local hospital computer. This caused significant problems with installation and upgrading working with doctors and IT-technicians from many centres in many countries. In the new software, the identifying data are stored encrypted on the central server or on a national server, but only the centre holds the de-cryption key to view the data, and they are not accessible in any way by the ECFSPR. In case the user loses the identifying data, they will have to re-enter them whereas the rest of the data will always be accessible.

##### Raw data and derived variables

Good data management practices impose that derived variables, such as BMI, are centrally computed by the DMU. The main reason for this is that, should an error in the computation occur, it would be easily traceable and recoverable. In order to minimise data-entry errors, it was decided to collect raw data (to which automatic plausibility checks are carried out) and delegate the computation task to the central DMU or to the software. This approach has the further advantage that the raw data can be transformed into standardised values (such as FEV1% of predicted, or height/weight standard deviation scores) according to different reference equations, as needed.

#### Data quality controls

Basic data quality controls should initially check that entered values lie within plausible ranges. Intra-record data coherence must be checked, such as chronological sequence of dates (e.g. the date of diagnosis has to follow the date of birth unless pre-natal diagnosis was performed) and consistent information (e.g. if values for a test are present, then the test must be recorded as “performed”) also across years (e.g. patients reported as liver transplanted one year should still be reported as transplanted throughout all subsequent years).

The ECFSPR has implemented a data quality control procedure composed of two levels: one carried out by the software for data entry and another carried out by the DMU.

##### First level of data quality controls (performed by the software)

For the manual data entry, correct coding is obtained by dropdown menus with fixed input possibilities (e.g. yes/no/unknown). The only field allowing free text is the mutation field: with more than 1900 known CF mutations, only the most frequent ones are included in a drop-down menu, with the possibility to manually enter new ones as free text. For numeric values, the format must be correct (e.g. integer, decimal, date), and the value should be within a pre-set range or it will not be accepted. Furthermore, values outside certain age-specific ranges will be flagged as possible errors, but will be accepted. For example, any value of height within the range 35–250 cm is accepted by the software, but a one-year old boy recorded as 100 cm high would be outside the age-specific range of 67–79 cm. His height value would therefore be accepted by the software but it will be flagged as a probable error to the user.

For the users who send the data through file upload we originally implemented the same controls, with upload denied for patients with unacceptable values and an error report of the problems. This could result in national registries not being able to finish uploading the full data set until errors had been corrected and re-uploaded. For this reason, we subsequently adopted another approach, described in the next section.

##### Second level of data quality controls (DMU)

The second step of data quality control procedure is identical for the two means of data sending and it is carried out after the annual data collection is closed. The DMU carries out more refined data plausibility and data coherence controls, for example, by using reference values for height and weight to detect potential errors by means of standard deviation scores, or by comparing values across years (e.g. decreasing values for height). The errors found are then uploaded onto the ECFSPR server as a file that points directly to the patient and the erroneous values with a short explanatory text. When opening the software, the users are led straight to the error and can either correct the value or confirm it; with a free text field available to send messages of explanation to the DMU.

When inconsistent data are found, data contributors should receive notification to revise data and send corrections within a pre-agreed short time frame. It is important that such notification is performed as quickly as possible. Mehta [14] reports that two weeks is a practical time limit by which centres should receive notification, because within this time people in charge of data-entry effectively remember the clinical notes and are able to retrieve the necessary information to correct the data. After this time, correcting the errors becomes less timely and efficient. This process becomes even more burdensome when requests of clarifications do not go directly to the centres that entered the data, but to intermediate data-management units (such as national registries).

For the centres manually entering the data, automatic data checks help avoid entry of wrong data, and since the patient’s file is usually available during data entry, the user can quickly find the correct value. In the new software we have added even further automated controls, diminishing the need for further corrections by the DMU.

For the national registries, however, the two-level data control turned out to be very inefficient. For a lot of the corrections requested, they need to contact the individual centres in their country – maybe even twice if errors were found in both levels of data control. This process could be very long and inefficient particularly if the national registries have already performed their own data quality controls and frozen the database for the analysis when the ECFSPR data collection starts. This is a specific issue for the ECFSPR, since we collect data with a delay of three years at the moment. The delay is primarily to allow the national registries to perform their data cleaning process and produce their own report before sending data to the ECFSPR. We aim to minimize the delay to one year. In order to optimize the error correction process a data quality control group, composed of national registry data managers and the ECFSPR team, has agreed on a common and exhaustive list of internal data checks to be carried out on the national databases by the national registries during their internal data cleaning process before data upload to the ECFSPR [22]. The national registries can now upload their checked data set without interruption and will just receive a list of any remaining questionable values.

After all the errors have been corrected by the data contributors, the data are frozen for analyses. Potential errors found by ECFSPR but not confirmed/corrected by the user, for the purposes of the annual data report, are deemed as erroneous and are set to missing.

### Handling of missing data

The amount of missing values has an important role on the interpretation of the results: if the data are missing for a non negligible portion of the database, then the estimates can be very imprecise or even biased if the underlying missing data mechanism is related to some specific values. For example, if 10% of the patients are reported to use insulin, but 20% of patients have unknown/missing information on use of insulin, the true percentage of use of insulin can be anything between 10 and 30%. For the annual ECFSPR data report we always report the number of missing data to illustrate the problem. The long experience of the ECFSPR team reveals that there are several reasons why missing data occur:

1. The lack of protected time, motivation and funding by a dedicated trained person in charge of data retrieval and data-entry is the biggest reason for the occurrence of missing data. Information not recorded at recruitment is rarely retrieved afterwards: data already entered are only occasionally revised by the CF centres, and their modification is performed only upon explicit request from the ECFSPR DMU. For example, some CF centres that send the data to the ECFSPR did not enter the information on the vital status of the patient (deceased/alive); when the ECFSPR DMU asked the centres if really the vital status of their patients was unknown, the centres answered that they did not have the time to enter this information, but that all the patients they reported were alive. The type of information requested and the time when the revision is requested are two critical points in the data correction process. There might be the need to consult the original clinical records and, sometimes, this can be problematic, especially if the time lapse is long. One way to reduce the workload for data entry is to automatically extract the information necessary for the registry from the computerised case report system that is used by a the CF centre. This has the advantage that if the data entered are used for clinical purposes, the centre operators are motivated to enter good-quality data for their own use. This solution was not applicable to the ECFSPR, due to the big heterogeneity of CF centres’ IT systems. With the implementation of the new data-entry software, though, we paid attention to create a user-friendly tool that could be used in clinical practice. Although it may not replace the software for patients management within the CF centres, the availability of a tool that allows users to see graphs of patient’s outcomes over time, centre data reports, and to download their centre’s data, hopefully will boost the motivation to actively participate to the ECFSPR by entering good quality data as well as reducing the amount of missing data;

2. The second reason for missing data is poor variables definition or misunderstandings in their coding. For example, the reason for some missing data on chronic infections in the last ECFSPR data collections was due to the fact that definition stated that culture was on sputum samples, and some centres interpreted this as only sputum and not cough swaps or other means of sampling, and hence left the variable as unknown if cultures were obtained by these other methods. The coding of liver disease also created missing values problems: the ECFSPR codes liver disease as (a) absence of liver disease, (b) presence of cirrhosis with hypertension, (c) presence of cirrhosis without hypertension, (d) presence of cirrhosis without specification of hypertension and (e) liver disease without cirrhosis. In the 2008–2009 data collection, one national registry collected and coded liver disease only as either presence or absence of cirrhosis with hypertension. This led to an incomplete classification of patients according to the ECFSPR criteria, because it was not possible to establish whether patients who did not have cirrhosis with hypertension were classifiable as code a, c, d or e;

3. The third reason for missing data is the poor specification of data fields: if a multiple-choice questionnaire does not provide the full range of possible answers, the operator that fills in the form can find it difficult to answer a question. A good way of avoiding this problem is to pilot the survey: sometimes the range of possible answers becomes clear only after answering the question in real settings. During such a pilot, we realised that recording information on neonatal screening test as positive or negative only was not exhaustive, because the test can have been performed and the result be positive, it can have been performed and the result be negative, it can have been performed and the result be unknown, it can have not been performed and it can be unknown whether the test has been performed or not. Thus we expanded the two original possibilities to five;

4. The fourth reason is the bad habit of answering a yes-no question only when there is an affirmative reply (sometimes aggravated by the software with only one tick box implying that an un-ticked box means no). This makes it hard to distinguish between true negatives (no), true unknowns (the user doesn’t know the information, so they deliberately leave the field untouched) and the omissions (the user forgot to answer the question: the answer could be either yes or no), as described by the insulin example above. From the beginning, the ECFSPR data collection form has required an active answer to all questions, without any pre-set values;

5. The fifth reason of the occurrence of missing data is the fact that not all the national registries collect the same variables, therefore some information is missing for some countries. For example, in the 2008–2009 data collection, for five countries the information on the use of inhaled hypertonic saline is missing because such information is not collected in a systematic way. In other cases the national registry definition is so different from the ECFSPR definition that the country chose to set the whole variable to missing, such as happened for one country with the information on chronic infection by *Pseudomonas aeruginosa*;

6. A final reason is the lack of information for particular sub-groups of patients due to specific patients’ characteristics. These are, for example, adults that do not have access to an adult CF centre and are lost to follow-up after leaving a children’s clinic, or transplanted patients who move from a CF centre to a transplant centre that does not participate to the registry. In the ECFSPR experience, there have been anecdotal reporting of the last group of patients from one national registry, but a thorough investigation of the problem has still to be carried out.

### Maintaining confidentiality of registry data

Anonymity of the individual patient is fundamental both in the patient’s decision to consent to their data use, and in the management of the data. This is particularly important in a rare disease where the number of patients is limited. Patient identifiers such as name, address and full date of birth are very rarely needed for the aims of any disease registry, as long as the patient can be identified for error correction by the patient’s care giver, who is privy to this information. Therefore, partial anonymisation (often called pseudo-anonymisation) can be used, whereby a patient ID can be linked to the full patient data only at the hospital site.

This poses other problems that need to be overcome: local handling of consents (because the signature on the consent form would disclose the patient’s name) and tailored software solutions (the local care giver must see the name of the patient while entering data to avoid confused identities, but the name cannot be sent to the central database).

The anonymity of data stored in the ECFSPR database was guaranteed by two means: access to person-identifiable data is granted only locally (CF centre or national registry) and creation of a random EU centre/country number so that the centre is not identifiable in the database. For national registries, no person identifiable data are transferred, but for the individual centres, who needed to see the patient name in order to enter the data correctly, these data are stored separately and encrypted without means of access from the ECFSPR team. The centres or national registries have all been issued a random centre. The link between the centre number and the centre name is held by the helpdesk personnel and the centre names do not appear in the registry database, thus enhancing anonymity also for patients attending very small centres.

### Dissemination of results and use of data

Timely dissemination of results and appropriate use of data collected are the key elements for achieving the registry gold objectives: enhance knowledge of the disease under study and promote research. There are several ways through which results can be disseminated: publication of technical reports, communication at conferences and publication in peer-reviewed journals. The use of data should be governed to avoid misuse of data. For this purpose the ECFSPR developed an initial set of guidelines, a code of conduct and terms of reference documents endorsed by the participating countries [23-25].

#### Annual reports

The ECFSPR annually publishes epidemiological descriptions of the data in a technical report, usually presented during the annual European Cystic Fibrosis Society Conference and subsequently available from the ECFSPR web site [26]. Frequency tables, descriptive statistics and graphs give updates on main aspects of CF epidemiology such as demographics, diagnosis, genetics, lung function, nutrition, microbiology, complications and mortality. Results are presented at European level and separately by country, to allow comparisons.

In the latest issue of the annual data report, a special effort was made to make the report contents more patient-friendly than in the previous issues: we used technical jargon only when necessary, we commented tables and graphs, we provided instructions on how to read more complex graphs (such as box-plots), and we added a glossary of medical and statistical terms. The report also features a section dedicated to patients, containing a message from the ECFSPR team and an invitation to send comments.

Another reporting activity, fundamental in a disease registry activity, is the feedback on the data given to the data contributors. This has the double advantage of improving data collection and empowering data contributors. The ECFSPR sends to the participating CF centres a customised report summarising the centre’s data and comparing them with data from other centres in the same country and with data from other countries. This gives the data contributor a report of the quality of the centre’s data and, most importantly, the status of their patients compared with other centres. Data comparisons are performed in an anonymous way: the centre names are not disclosed, safeguarding confidentiality of each centre’s aggregated data.

#### Peer-reviewed publications

Publication in peer-reviewed journals is probably the most efficient and effective way to share with the scientific community the findings of research and submit it to its scrutiny.

The scientific activity of the ECFSPR allowed, for example, enhancing knowledge on potential risk factors of pulmonary disease in CF patients, highlighting the importance of their early identification and timely intervention [27].

The availability of a large database, such as the ECFSPR, offers a unique opportunity to analyse data from different populations. This opportunity was fully exploited to provide the CF specialists with a useful additional tool for patients care: Boëlle et al. [28] published reference percentiles for FEV1 and BMI derived from a CF population. This allows CF specialist to have additional information on the patients they care for by comparing their lung function and BMI with their CF peers instead of against a healthy population alone.

Another important area of research activity is providing information for therapy development. This fundamental contribution was achieved in another publication [29], where the authors describe the CFTR mutation class spectrum across Europe, highlighting which are the mutation classes to target for drug development in order to maximise the number of patients that will benefit from it and pointing out in which countries clinical trials could be performed thanks to the availability of patients carrying specific mutations.

Finally, research outcomes have the potential to urge for political decisions that have an impact on CF patients’ life. The paper by McCormick et al. [9] is an example of how comparison of simple demographic indicators highlights health care inequalities: the authors in fact showed that very different age structures of CF patients were observed between countries, despite a common genotype, according to their longevity of European Union membership, suggesting that health-care spending in new EU entrant countries would correspond to improved survival of patients.

#### Access to data

Making the data available to the scientific community for research purposes is a noble and a burdensome responsibility. Granting access entails the responsibility to provide high quality data and ensuring legal and ethical use of the data by third parties.

The ECFSPR decided to grant access of the data only after some years of its activity, when crucial issues on uniformity of definitions and data completeness had been resolved. Access is strictly regulated by a Standard Operating Procedure, according to which requests on the use of data are reviewed by a scientific committee that formulates a recommendation for its approval and the request is forwarded to all the data contributors that ultimately give permission to use their data. A clear authorship, acknowledgement and publication policy has been set up, to ensure fair recognition of people’s work and contribution [24]. The data collected by the ECFSPR are at the moment being analysed in order to estimate the need for and plan the care of adult CF patients in the future (ERS/ECFS Task Force on Adult Care in Cystic Fibrosis).

## Discussion

There are some critical points in a disease registry development that are independent of resource allocation and that should be carefully considered in the planning phases of a disease registry.

The first important point is the definition of the information to record. The experience gained by the ECFSPR definitions working group proved that the most cost-effective approach is to first work on the definition of a few variables, then pilot the definition for data collection on a restricted group of data contributors or for one data collection point, make the necessary amendments and then move on with the definition of other variables. This sequential approach, as opposed to the one of completing the full set of definitions in one go, has the advantages of not delaying the data collection for too long: if definitions are agreed on a core set of variables of high research interest first, data collection can start straight away, instead of being postponed by the time needed to define other, less important variables. The initial ECFSPR pilot study highlighted the importance of having common definitions for the variables to collect. If we had started data collection before already established databases from national CF registries, fewer compromises would have been necessary on definitions and on the level of detail of information collected, but such an opportunity is probably not available for most rare diseases. In order to set up definitions that would make data analyses results useful and at the same time data retrieval from clinical files feasible, we had to sacrifice some of the internationally acknowledged definitions; in some cases, to avoid large amount of missing data, we had to use proxy variables, such as use of pancreatic enzymes as a proxy for pancreatic insufficiency in order to guarantee fair comparisons across countries. In the most critical cases, national registries recorded information according to internally-agreed definitions, and where compromise was not feasible we had to content ourselves with missing data from those countries where their definitions were too far from what we intended. Fortunately, the national registries have been very collaborative, sometimes even changing their definitions to accommodate the ECFSPR ones in order to reduce the amount of missing data. Involving the national registry representatives in the definitions workgroup has been crucial for this understanding. For this reason we encourage researchers willing to set up disease registries to have early meeting with all potential data contributors, and to start a collaborative relationship. Finally, the ECFSPR definitions group advised a periodic revision of the information registered. This task is considered fundamental for an efficient perspective data collection, for three reasons: (1) definitions need often to be validated in real settings, and in some cases clarifications are necessary to people in charge of data retrieval and data recording; (2) variables might prove to be of limited utility for research due to the way they have been defined or coded; (3) improvements in knowledge of the disease and advances in scientific/technological discoveries make the collection of new information necessary: the registry needs to be constantly in tune with changes, to avoid that the information collected is no longer useful to researchers and clinicians. The ECFSPR is starting the second revision of its data collection form, reviewing the definition of some variables (such as diabetes) and evaluating the inclusion of new ones (e.g. computerised tomography imaging and lung clearance index). It is important to remember that such revision and the choice to modify the information collected has an impact on the data already collected: careful examination of whether there will be limitations in linking the data across the years should be performed. Similarly, when choosing to add new information to data collection, the effort needed to retrieve such information for the patients already included in the registry has to be carefully evaluated. For these reasons, the ECFSPR will have to carefully balance the advantages and disadvantages of modifying the data-entry forms.

Although adoption of common definitions, together with the use of a common data collection platform, should guarantee comparable data across countries, differences in outcomes between countries can still be observed, and there is a risk of over- or mis-interpreting these. They can be due to many factors, like different population demographics, health care systems, standards of care and national economics. These are the true differences that should be discussed and used for improvement of CF outcomes. However, differences may also be due to measurement methods and different translation of clinical findings: observational studies (such as patient registries) are more prone than other study designs (such as clinical trials) to the risk of artificial differences due to e.g. different measurement instruments or measurement practices. When such heterogeneity is observed, it is advisable that the registry validates the data to ensure that the differences seen are real. For example, in the ECFSPR 2008–2009 data collection, we found bigger differences in liver disease severity than expected from the natural history of this complication. For this reason the ECFSPR recently started a project on liver disease investigating the diagnostic work-up performed on randomly chosen patients and centres participating to the ECFSPR. Also, the ECFSPR decided to use internationally agreed reference values for pulmonary function and anthropometric measurements, but there is the awareness that such choice is not appropriate for all the CF populations registered in the ECFSPR, therefore standardised values are used only when comparison between countries are carried out, and careful comments always accompany the outcomes of such comparisons.

Another crucial aspect in a disease registry development is the careful consideration of the growing registry needs. With 23 countries, almost as many languages and the reality of working with both national registries with more than 5,000 patients and small centres with less than 50 patients, the ECFSPR had to face many challenges. The choice of an electronic data collection system eased the data-recording process, but it triggered the recruitment of additional workforce (IT experts, helpdesk staff for technical assistance) and the need of technical devices (e.g. server) that need maintenance. The software for data-entry must fulfil standards of quality and security, it should be tailored to the needs of the registry, and it should be user-friendly. Ideally, the physicians should use the data collected through the software in the daily patient management, thus rewarding them directly for the data retrieval and data-entry effort. The patients that consented to have their data collected should be given graphical feedback through the software, to see the benefits of participation to the registry. The first software adopted by the ECFSPR was quite demanding in terms of on-site installation and maintenance, and the burden on the centres as well as the technical helpdesk was considerable. This was an important lesson learned for the development of the new software. A multilingual helpdesk approach has been necessary even if the official language of the registry is English. The development of standard operating procedures, the drafting of technical documentation, and the use of document-sharing technologies and web teleconferencing have proven very effective in the daily management of the operational activities of the ECFSPR. This increased complexity in the registry structure has to be carefully considered by researchers, and an accurate cost-benefit analysis should be performed, especially when the funding opportunities are not secured. At the moment, the ECFSPR employs one full time coordinator, one full time statistician and two part-time help-desk personnel. Additional cost of software development will vary depending on the need. Further to this, the cost of running the national registries may be even higher in the larger countries, whereas some smaller countries rely almost entirely on volunteer effort by doctors or patient organisations. It is crucial for the sustainability of the registry, that these costs are compared to the possibility of funding.

One future aspect of the ECFSPR is to expand cooperation with the pharmaceutical industry and EMA in order to collect pharmacovigilance data on new drugs. This work is in progress in some of the national registries (such as UK and Germany), but until now the ECFSPR has been hampered by the three year delay in data collection, which would not allow timely identification of serious side effects. However, with the new software, which allows real-time use in the clinical setting, the possibility of pharmacovigilance data collection is open for the centres and countries reporting directly to the ECFSPR.

Finally, missing data is a well-known problem for the registries without a unique solution. Often missing data can be avoided by appropriate choice of information to be recorded, adequate level of detail and coding of information, and availability of well-trained, motivated and dedicated personnel in charge of data retrieval and data entry. The ECFSPR collects data from national registries that for some centres have funded data entry, and for others require data entry in order to be acknowledged as a CF centre. These are ways to motivate people, but at the moment, the ECFSPR does not have funding for the participating centres (and neither any authority over them). Another way to motivate people is to show them the utility of the data they have been collecting at regular feedback sessions as we do with the annual report and the centre report; and lately with the new software that offers interactive graphs and tables for use in the daily clinic management and for patient information.

## Conclusions

Setting up and maintaining a disease registry is a burdensome venture. Since the tentative beginning in 2003, the ECFSPR has evolved from a small working group of enthusiastic and knowledgeable national registry representatives, collecting data on spreadsheet files with very little funding and resources, to a professionally run patient registry with full and part time dedicated personnel that includes an executive director, an executive coordinator, two statisticians, an helpdesk service, a governing body composed by ten people (Executive Committee), a bespoke software and extensive use of data collected from 23 countries and more than 30,000 patients. The road to this has been paved with obstacles and challenges, and the journey is by no means at an end. A registry of these proportions may be initiated, but cannot be run as an amateur project by a few dedicated people; we could have probably accomplished our goals faster and have had fewer bumps on the road had the funding been in place at an earlier stage. For this reason, we recommend that objectives, structure and research outcomes are planned according to the available funding, in order to optimise resources allocation and avoid early frustration.

In the ECFSPR experience we found crucial for success the collaboration with existing national and international registries and cystic-fibrosis organisations (such as the ECFS Clinical Trial Network). Particularly helpful has been the patients’ involvement in the registry activities through their representatives in governance committees in order to better meet the patients’ needs and to convey the information about the registry in an effective way (through the patient-friendly report and the publication of web pages dedicated to patients in the ECFSPR website).

Another key aspect has been the networking for the recruitment of new centres to contribute the data, and the stimulation of their participation through their empowerment (participation to the ECFSPR governance bodies), through fair reward by co-authorship in peer-reviewed publications, and by publication of periodical (centre-based) data reports.

Finally, funding is a pivotal aspect in a disease registry running. The current registry sustainability cost is in staff (100,000 Euros per year) to retrieve, check, and analyse the data. But since the real cost to set up the registry exceeded 1,000,000 Euros over the last decade, this experience creates a cost-effective approach because the experience is donated as a gift to the community. A system for centres accreditation and funding according to centre’s data completeness and data quality has been successfully used in many disease registries. This approach, however, is quite ambitious for most registries, especially for rare diseases. Pragmatically, where funding is limited, much can be achieved by restricting the data collection to a core set of data, usually referring to demographic, diagnosis and death information, which are easy to retrieve for most data contributors [9]. The cost of running a national registry varies depending on the size and organisation of the national registry, and whether it is used locally for e.g. quality control. The new ECFSPR software will offer a cheaper solution for some countries by supplying free software and data availability locally and nationally.

## Abbreviations

CF: Cystic fibrosis; DMU: Data management unit; ECFSPR: European cystic fibrosis society patient registry; ECFS: European cystic fibrosis society; XML: Extensible markup language; FEV1: Forced expiratory volume in 1 second.

## Competing interest

The authors declare that they have no competing interest.

## Authors’ contributions

LV conceived, wrote and reviewed the manuscript. AZ contributed to writing and reviewed the manuscript. AM reviewed the manuscript. HVO contributed to writing and reviewed the manuscript. All authors read and approved the final manuscript.
